# Genetic Analysis of the Lambda Spanins Rz and Rz1: Identification of Functional Domains

**DOI:** 10.1534/g3.116.037192

**Published:** 2016-12-28

**Authors:** Jesse Cahill, Manoj Rajaure, Chandler O’Leary, Jordan Sloan, Armando Marrufo, Ashley Holt, Aneesha Kulkarni, Oscar Hernandez, Ry Young

**Affiliations:** Center for Phage Technology and Department of Biochemistry and Biophysics, Texas A&M University, College Station, Texas 77843

**Keywords:** lysis, phage, *Escherichia coli*, membrane fusion

## Abstract

Coliphage lambda proteins Rz and Rz1 are the inner membrane and outer membrane subunits of the spanin complex—a heterotetramer that bridges the periplasm and is essential for the disruption of the outer membrane during phage lysis. Recent evidence suggests the spanin complex functions by fusing the inner and outer membrane. Here, we use a genetics approach to investigate and characterize determinants of spanin function. Because *Rz1* is entirely embedded in the +1 reading frame of *Rz*, the genes were disembedded before using random mutagenesis to construct a library of lysis-defective alleles for both genes. Surprisingly, most of the lysis-defective missense mutants exhibited normal accumulation or localization *in vivo*, and also were found to be normal for complex formation *in vitro*. Analysis of the distribution and nature of single missense mutations revealed subdomains that resemble key motifs in established membrane-fusion systems, *i.e.*, two coiled-coil domains in Rz, a proline-rich region of Rz1, and flexible linkers in both proteins. When coding sequences are aligned respective to the embedded genetic architecture of *Rz1* within *Rz*, genetically silent domains of *Rz1* correspond to mutationally sensitive domains in *Rz*, and vice versa, suggesting that the modular structure of the two subunits facilitated the evolutionary compression that resulted in the unique embedded gene architecture.

For Caudovirales, host lysis is essential for the release of phage progeny. For Gram-negative hosts, lysis is a three-step process requiring the function of three types of phage-encoded proteins: holins, endolysins, and spanins (Berry *et al.* 2008; Savva *et al.* 2014).

The first two steps, in which the holin permeabilizes the inner membrane (IM), and the endolysin degrades the peptidoglycan (PG), have been studied intensively for decades, and were considered necessary and sufficient for host lysis. However, recently we have found that lysis requires the disruption of the outer membrane (OM) by phage-encoded spanins (Young 1992; Berry *et al.* 2012). In lambda, the spanin complex is composed of two subunits: Rz, an IM protein, or i-spanin and Rz1, an OM lipoprotein, or o-spanin. The Rz1 gene is encoded in a bizarre genetic architecture, embedded in the +1 reading frame of *Rz* (Figure 1, A–C). Rz has type-II IM topology (N-in, C-out), with a single N-terminal transmembrane domain (TMD) and a periplasmic domain (Berry *et al.* 2008, 2010). Rz1 is a lipoprotein, anchored in the inner leaflet of the OM (Kedzierska *et al.* 1996; Berry *et al.* 2008). Rz and Rz1 interact via their periplasmic domains, forming a complex that spans the entire periplasm (thus the term spanin) (Berry *et al.* 2008). A defect in either spanin subunit blocks lysis, leaving spherical cells bounded by the intact OM (Berry *et al.* 2012). Two-component spanin equivalents are present in nearly all Caudovirales of Gram-negative hosts. In a minority of cases, spanin function is fulfilled by a single protein, the unimolecular spanin or u-spanin. The u-spanin is anchored to the inner leaflet of the OM by a lipoylated N-terminal Cys residue, and embedded in the IM by a C-terminal TMD anchor (Summer *et al.* 2007).

**Figure 1:**
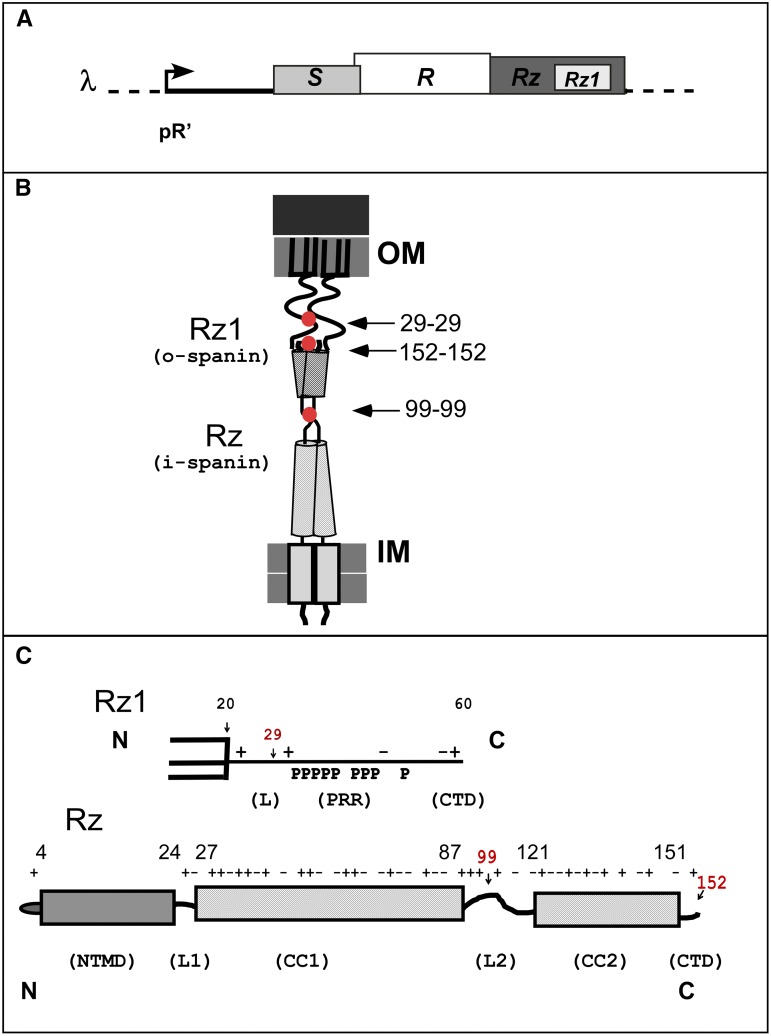
Features of the lambda spanins. (A) The lambda lysis cassette. The lysis genes of lambda are S, R, and Rz/Rz1, and are under control of the late promoter pR’. (B) Cartoon of the spanin complex in prehairpin conformation within the cell envelope. The lambda o-spanin, Rz1, is a dimer that is anchored to the inner leaflet of the OM by a lipid anchor. The lambda i-spanin, Rz, is a dimer anchored to the IM by a TMD. Rz extends two helices into the periplasm. Red dots and arrows denote the location of the homotypic intermolecular disulfide bonds. (C) Cartoon schematic of Rz1 and Rz, highlighting charge distribution, predicted secondary structure features, and domains. The positions of Cys residues are indicated in red.

Molecular characterization of lambda spanins revealed that both Rz and Rz1 accumulate as homodimers linked by homotypic intermolecular disulfide bonds: both of the Cys residues (C99 and C152) in Rz are involved in these bonds, as is the single Cys residue (C29) in Rz1 (Figure 1B) (Berry *et al.* 2013). Although the Rz disulfide linkage at C99 is irrelevant, spanin function requires either the Rz C152 or the Rz1 C29 homotypic disulfide linkage. In the absence of Rz1 or in the *Rz_C152S_Rz1_C29S_* double mutant, Rz undergoes substantial proteolytic cleavage. This indicates that complex formation is required for stabilization of Rz in the periplasm, and suggests covalent homodimerization of the spanin subunits has a role in complex formation.

Rz1 was predicted to be largely unstructured due to its high proline content (10 prolines in the 40 aa mature Rz1). By contrast, the periplasmic domain of Rz was predicted to be highly structured, dominated by two coiled-coil helical domains (Figure 1C) (Berry *et al.* 2010). Circular dichroism (CD) studies of the purified periplasmic domains supported these predictions (Berry *et al.* 2010).

Recently, it was demonstrated that Rz and Rz1 can mediate fusion between two membrane bilayers, suggesting a general model in which the disruption of the OM is topological, *i.e.*, reflecting the fusion of the IM and OM (Rajaure *et al.* 2015). It has been proposed that, after PG degradation, spanin complexes undergo a conformational change that brings the opposing membrane bilayers into close proximity for fusion (Rajaure *et al.* 2015). Evidence for major conformational dynamics was provided by CD analysis, which showed that mixing the periplasmic domains of Rz and Rz1 *in vitro* resulted in the formation of rod-like bundles, and a large increase in helical content (Berry *et al.* 2010). We have suggested that the coiled-coil domains participate in this conformational change, as has been shown for well-studied membrane fusion systems in eukaryotes, including the influenza virus HA2 fusion protein (Weber *et al.* 1998).

Lysis proteins are inherently intractable to biochemical and structural analysis, primarily due to being membrane-embedded and highly oligomeric. Thus, exhaustive genetic screens are required to increase the mechanistic understanding of other lysis protein components, such as holins and pinholins (Ramanculov and Young 2001; Pang *et al.* 2010; Gründling *et al.* 2000). We wanted to use phage genetics to address spanin function, with the goal of isolating mutants blocking intermediate steps in the lytic pathway. A comprehensive genetic analysis of the lambda spanins has not been attempted, mostly due to the embedded genetic architecture of *Rz1* within of *Rz* (Figure 1A). Here, we report the results of a nearly saturating mutagenesis of the lambda spanins. The results are discussed in terms of spanin function, postulated intermediate steps of the Rz-Rz1 membrane fusion pathway, and evolution of the embedded gene architecture.

## Materials and Methods

### Bacterial strains, plasmids, bacteriophages, and growth and induction conditions

The bacterial strains, bacteriophages, and plasmids used in this study are described in Table 1 and Table 2. Bacterial cultures were grown in standard LB medium or, as appropriate, in LBM, which is supplemented with MgCl_2_ (10 mM). When appropriate, ampicillin (Amp, 100 μg ml^−1^) and kanamycin (Kan, 40 μg ml^−1^) were also added. Growth and lysis of cultures were monitored by A_550_ as a function of time, as described previously (Berry *et al.* 2012). Lysogenic cultures were thermally induced at A_550_ ∼0.2 by a shift to 42° for 15 min, followed by continued growth at 37°. For inductions of nonlysogenic cultures, isopropyl β-d-thiogalactopyranoside (IPTG) was added to the final concentration of 1 mM for plasmid induction. The pRE plasmid is a pBR322 derivative, which has the lambda late promoter pR’ located upstream of the *Rz* or *Rz1* start site. To activate pR’, the antiterminator Q is supplied *in trans* by either the induced prophage or the pQ plasmid.

**Table 1:** Phages, strains, and plasmids used in this study

		Genotypes and Relevant Features	Sources
Bacteriophages			
λ900		λΔ*(stf tfa)*::*cat cI_857_ bor*::*kan*; carries Cam^R^ and Kan^R^	Laboratory stock
λ900*Rz_Q100am_ Rz1^+^*		*Rz* gene carries amber codon at position 100 with silent change in embedded *Rz1*	Laboratory stock
λ900*Rz^+^Rz1_W38am_*		*Rz1* gene carries amber codon at position 38 with silent change in overlapping *Rz*	Laboratory stock
λ900*Rz_Q100am_ Rz1_W38am_*			Laboratory stock
Strains			
MC4100*tonA*::*Tn10*		*Escherichia coli K-12 F araD139* Δ*(argF-lac)U169 rpsL15 relA1 flbB3501 deo pstF25 rbsR tonA*	Laboratory stock
MC4100 λ900		MC4100 *tonA*::*Tn10* lysogenized with λ900	Laboratory stock
MC4100 λ900*Rz_Q100am_ Rz1^+^*		MC4100 *tonA*::*Tn10* lysogenized with λ900 *Rz_Q100am_ Rz1^+^*	Laboratory stock
MC4100 λ900*Rz^+^Rz1_W38am_*		MC4100 *tonA*::*Tn10* lysogenized with λ900 *Rz^+^Rz1_W38am_*	Laboratory stock
MC4100 λ900*Rz_Q100am_ Rz1_W38am_*		MC4100 *tonA*::*Tn10* lysogenized with λ900 *Rz_Q100am_ Rz1_W38am_*	Laboratory stock
RY17341		MDS12Δ*tonA*; MG1655 with 12 deletions, totaling 376,180 nt, including cryptic prophages	Laboratory stock
RY17341 λ		RY17341 lysogenized with temperature sensitive λcI857	This study
RY17299 *lacI^q1^*		Derived from MG1655 Δ*tonA*	Park *et al.* (2006)
Plasmids			
pRE		Plasmid with the λ later promoter pR’ that is transcriptionally activated by λQ	Laboratory stock
pRz		pRE carrying Rz alone with Rz1 inactivated by a nonsense mutation	Laboratory stock
pRz1		pRE carrying Rz1	Laboratory stock
pRz mutX		pRE carrying denoted mutation of Rz	This study
pRz1 mutX		pRE carrying denoted mutation of Rz1	This study
pSynRz		pRE carrying Rz alone. The former region of Rz1 overlap within Rz was codon-optimized to be genetically disparate from WT Rz1 to avoid recombination	This study
pSynRz1		pRE carrying Rz1 alone. SynRz1 was codon-optimized to be genetically disparate from WT Rz to avoid recombination	This study
pSynRz mutX		pRE carrying denoted mutation within SynRz	This study
pSynRz1 mutX		pRE carrying denoted mutation within SynRz1	This study
pSynRz Linker 101–115		Residues 101–115 of Rz replaced with Gly-Ser repeats	
pLinkerRz Q100S		Residues 100–115 of Rz replaced with eight Ser-Gly repeats	This study
pRz1 25–30 GS-Linker		Residues 25–30 of Rz1 replaced with 3 Gly-Ser repeats	This study
pRz1_his_		Rz1 with His-tag at the C- terminal end	Berry *et al.* (2008)
pQ		pSC101 origin with low-copy mutation; *Q* cloned under P_lac/ara-1_ promoter	Gründling *et al.* (2001)

**Table 2:** Primers used in this study

Primer	Sequence 5′–3′
pRz S20P FOR	TCTGCCTGCCATGGGCTGTTAATC
pRz S20P REV	GATTAACAGCCCATGGCAGGCAGA
pRz Q36P	GAGATAACGCCATTACCTACAAAGCCCCGCGCGACAAAAATGCCAGAGAAC
pRz A50P	CTGAAGCTGGCGAACGCGCCAATTACTGACATGCAGATGCGTCAGC
pRz A62P	CAGATGCGTCAGCGTGATGTTCCTGCGCTCGATGCAAAATACACGAAG
pRz T107P FOR	GTGAAGCCACCCCCGCCTCCGGCGTAGATAATG
pRz R125P	CTGGCAGACACCGCTGAACCGGATTATTTCACCCTCAGAGAGAGGC
pRz E150G	CAACTGGAAGGAACCCAGAAGTATATTTAGGAGCAGTGCAGATAGGGATCC
pRE Rz Q151P	CCAGAAGTATATTAATGAGCCGTGCAGATAGGG
pRz Q151X	AACAACTGGAAGGAACCCAGAAGTATATTAATGAGTAATGCAGATAGGGATCCGTCGAC
pRz C152X	CTGGAAGGAACCCAGAAGTATATTAATGACCAGTAAAGATAGGGATCCGTCGACCTGC
pRz R153X	GGAACCCAGAAGTATATTAATGAGCAGTGCTAATAGGGATCCGTCGACCTGCAG
pSynRz Linker 101–115 FOR	CGGCAGTGGTAGTGGTAGTGGAAGTCCACGGCTAGCGGAT
pSynRz Linker 101–115 REV	CTGCCGCTGCCACTACCGCTTCCTTGGCAAACCGCCTTAATATG
SynRz E150R SynRz1 R59E*	CCCTTAGGTACCAGAGAGATTGATGTATGAGCAGAGTCACCGCGATTATCTCCGCTCTGGTTATCTGCATCATCGTCTGCCTGTCATGGGCTGTTAATCATTACCGTGATAACGCCATTACCTACAAAGCCCAGCGCGACAAAAATGCCAGAGAACTGAAGCTGGCGAACGCGGCAATTACTGACATGCAGATGCGTCAGCGTGATGTTGCTGCGCTCGATGCAAAATACACGAAGGAGTTAGCTGACGCCAAGGCGGAGAACGACGCGCTACGGGACGACGTGGCAGCCGGGCGGCGCCGATTACATATTAAGGCGGTTTGCCAATCCGTACGGGAGGCTACTACAGCAAGTGGAGTAGACAACGCGGCAAGTCCACGGCTAGCGGATACTGCCGAGCGAGACTACTTTACACTTAGGGAAAGACTAATCACTATGCAAAAACAACTGGAAGGAACCCAGAAGTATATTAATAGGCAGTGCAGATAGGGATCCAAGGAGTTAGCTGATGCTTAAACTCAAGATGATGCTATGTGTAATGATGTTACCACTTGTTGTAGTTGGGTGTACGAGTAAACAATCGGTATCGCAATGTGTAAAACCGCCCCCACCGCCTGCATGGATCATGCAACCGCCACCTGATTGGCAAACGCCACTAAATGGAATCATATCGCCATCGGAAGAGGGATGAAAGCTTCTGTTTTG
pRz_ART-TMD_**	ATATGGTACCAGAGAGATTGATGTATGAGCAGAGTGGTGCTGCTGATTATTGTGGTGGTGGTGGTGGTGGTGGTGATTATTCTGCTGATTATTGTGCATTACCGTGATAACGCCATTACCTACAAAGCCCAGCGCGACAAAAATGCCAGAGAACTGAAGCTGGCGAACGCGGCAATTACTGACATGCAGATGCGTCAGCGTGATGTTGCTGCGCTCGATGCAAAATACACGAAGGAGTTAGCTGACGCCAAGGCGGAGAACGACGCGCTACGGGACGACGTGGCAGCCGGGCGGCGCCGATTACATATTAAGGCGGTTTGCCAATCCGTACGGGAGGCTACTACAGCAAGTGGAGTAGACAACGCGGCAAGTCCACGGCTAGCGGATACTGCCGAGCGAGACTACTTTACACTTAGGGAAAGACTAATCACTATGCAAAAACAACTGGAAGGAACCCAGAAGTATATTAATGAGCAGTGCAGATAGGGATCCGCG
pLinkerRz Q100S	GCCGATTACATATTAAGGCGGTTTGCTCAGGAAGCGGTAGTGGCAGC
pRz1 25–30 GS-Linker FOR	TCCGGCTCCAAGCCACCACCGCCTCCG
pRz1 25–30 GS-Linker REV	GCCGGAGCCCTGCTTTGATGTGCAACCGAC
pSynRz1 D45K FOR	AACCGCCACCTAAATGGCAAACGCC
pSynRz1 D45K REV	GGCGTTTGCCATTTAGGTGGCGGTT
pSynRz1 I54X	GATTGGCAAACGCCACTAAATGGAATCTAATCGCCATCGGAAAGGGGATG
pSynRz1 S55X	GGCAAACGCCACTAAATGGAATCATATAGCCATCGGAAAGGGGATGAAAGC
pSynRz1 P56X FOR	GGAATCATATCGTGATCGGAAAGGGG
pSynRz1 P56X REV	CCCCTTTCCGATCACGATATGATTCC
pSynRz1 S57X FOR	CAATCATATCGCCATAAGAAAGGGGATGAAAG
pSynRz1 S57X REV	CTTTCATCCCCTTTCTTATGGCGATATGATC
pSynRz1 E58X	CCACTAAATGGAATCATATCGCCATCGTAAAGGGGATGAAAGCTTCTGTTTTG
pRz1-His P32Q FOR	CGTGAAGCAACCACCGCC
pRz1-His P32Q REV	GGCGGTGGTTGCTTCACG
pRz1-His P33L FOR	GTGAAGCCACTGCCGCCTCCGGCG
pRz1-His P33L REV	CGCCGGAGGCGGCAGTGGCTTCAC
pRz1-His P35H FOR	CCACCACCGCATCCGGCGTGG
pRz1-His P35H REV	CCACGCCGGATGCGGTGGTGG
pRz1-His P36Q FOR	CCACCACCGCCTCAGGCGTGGATAATG
pRz1-His P36Q REV	CATTATCCACGCCTGAGGCGCTGGTGG
pRz1-His I39V FOR	CCGGCGTGGGTAATGCAGC
pRz1-His I39V REV	GCTGCATTACCCACGCCGG
pRz1-His P44S FOR	CAGCCTCCCTCCGACTGGC
pRz1-His P44S REV	GCCAGTCGGAGGGAGGCTG
pRz1-His W46R FOR	CCCCCCGACCGGCAGACAC
pRz1-His W46R REV	GTGTCTGCCGGTCGGGGGG
pRz1-His L50P FOR	CAGACACCGCCGAACGGGATTATTTC
pRz1-His L50P REV	GAAATAATCCCGTTCGGCGGTGTCTG
pRz1-His R59E FOR	ATTTCACCCTCAGAGGAAGGCGGCCAC
pRz1-His R59 REV	TGGCCGCCTTACTCTGAGGGTGAAATAATCC

The “*” and “**” symbols indicate a dsDNA gblock (Integrated DNA Technologies) synthesized gene (Genscript) designed with a spanin allele flanked by restriction sites compatible with the pRE plasmid.

### Error-prone PCR mutagenesis and selection for lysis-defective Rz and Rz1

Error-prone PCR mutagenesis was performed using the GeneMorph II random mutagenesis kit without any modification to the manufacturer’s instructions. To maximize the single nucleotide changes, pRz or pRz1 template DNA of higher concentration (∼5 μg) was used. The Rz1 gene is inactivated on the pRz plasmid by a nonsense mutation that is silent in *Rz* (Table 1 and Table 2). Oligonucleotides were obtained from Integrated DNA technologies (Coralville, IA).

Mutagenized PCR products were digested with *Kpn*I and *Bam*HI for *Rz*, and *Bam*HI and *Hin*dIII for *Rz1*. The gel-purified, doubly digested, fragments were ligated into the pRE vector using T4 ligase, and transformed into XL-1 Blue cells. After overnight incubation at 37°, the transformants were pooled by slurrying, and plasmid DNA was extracted using the Qiagen spin miniprep kit. MC4100 (λ) lysogens carrying the nonsense alleles *Rz_Q100 am_* or *Rz1_W38 am_* were transformed with the mutagenized plasmid pool of *Rz* or *Rz1*, respectively. To assess the frequency of mutation, 10 random colonies from each library were tested for lysis defects in liquid culture, and their spanin genes sequenced. Six *Rz* plasmids and four *Rz1* plasmids did not complement the lysis-deficient phenotype. Of the remaining clones, three of the latter had missense changes that did not abrogate lysis phenotype. To enrich for lysis-defective mutants, a plasmid retention method was used. Colonies on the transformant plate were collected by slurring with LB, diluted and inoculated into 25 ml of LBM and appropriate antibiotics at an initial A_550_ ∼0.5, induced for lysis as described above. At 15 min past the normal lysis time (∼50 min), the culture was centrifuged at 4000 rpm for 5 min to harvest the nonlysed, Mg^++^-stabilized, spherical cells, the terminal phenotype of spanin-defective lysis. The harvested culture was carefully washed once with LBM before extracting plasmid DNA using a miniprep kit. Plasmids from the enriched mutant pools of *Rz* and *Rz1* plasmids were used to transform λ lysogens of *Rz_am_* and *Rz1_am_*, respectively. Single colonies were picked and individually screened for a lysis defect by thermal induction in 5 ml LBM. Lysis-defective clones were sequenced by Eton Biosciences (San Diego, CA).

### Detection and quantification of spanin proteins

Accumulation of *Rz* or *Rz1* gene products was assessed by Western blotting of TCA precipitates as described previously (Berry *et al.* 2013). Briefly, lysogens with *Rzam* or *Rz1 am* mutations were transformed with the pRE plasmid carrying an *Rz* or *Rz1* allele. ∼50 min after induction, a 1 ml aliquot was precipitated with 10% TCA (Berry *et al.* 2012). Samples were normalized to A550 units, and resolved on a 16.5% SDS-PAGE gel. When needed, the His-tagged proteins were probed using anti-His antibody from Sigma-Aldrich.

### Covariance analysis

We identified a lambda family of embedded two-component spanin equivalents based on 40% sequence similarity over 40% of sequence length (R. Kongari and R. Young, unpublished data). From this family, we selected six representatives of the i- and o-spanin C-terminal domains.

### Identification of codons a single base pair change from Pro

Using Python and BioPython (Chapman and Chang 2000), we developed the tool One SNP Away to scan a FASTA sequence for codons that are a single nucleotide change from the query amino acid. This program was used here to identify codons that could be mutated to proline via one mutational step. This tool is available on the Center for Phage Technology’s Galaxy Instance (https://cpt.tamu.edu/galaxy-pub/).

### Data availability

Strains and reagents are available upon request.

## Results and Discussion

### Design and implementation of the mutagenesis system

Plasmids carrying Rz or Rz1 (pRz and pRz1) under the native pR’ late promoter were used to effect independent mutational analysis on each gene. Both pRz and pRz1 complemented the lysis defect of lambda *Rz_am_* or lambda *Rz1_am_*, respectively (see *Materials and Methods*). The spanin genes were then subjected to random PCR-based mutagenesis in the context of these complementing plasmids, subcloned into a fresh vector, and transformed into a host carrying the *Rz_am_* or *Rz1_am_* lysis-defective prophage. The pooled transformant libraries of pRz and pRz1 were selected for lysis-defective alleles by plasmid retention (see *Materials and Methods*), and then screened individually for lysis after induction. A total of 266 pRz and 115 pRz1 lysis-defective clones were sequenced, of which 131 *Rz* and 79 *Rz1* genes had single point mutations. The remaining lysis-defective *Rz* and *Rz1* mutants had either multiple point mutations (48 *Rz* and 19 *Rz1*) or frameshifts, including both base insertions and deletions (87 *Rz* and 17 Rz1). Overall, the nucleotide changes were 42% transition and 58% transversion mutations, as expected for this type of mutagenesis, which indicates that the full range of possible mutations at each codon was equally accessible (Hanson-Manful and Patrick 2013).

### Mutational analysis of Rz

For convenience and to emphasize structural features, the Rz sequence (Figure 1C) is subdivided into six domains: the N-terminal TMD domain, NTMD (residues 1–24); the two parallel coiled-coil domains, as predicted by COILS software (Lupas *et al.* 1991) CC1 (residues 27–87) and CC2 (residues 121–150), linkers, L1 and L2, separating the aforementioned domains, and an extreme C-terminal domain (CTD).

#### Lysis-defective nonsense mutants of Rz:

**Figure 2:**
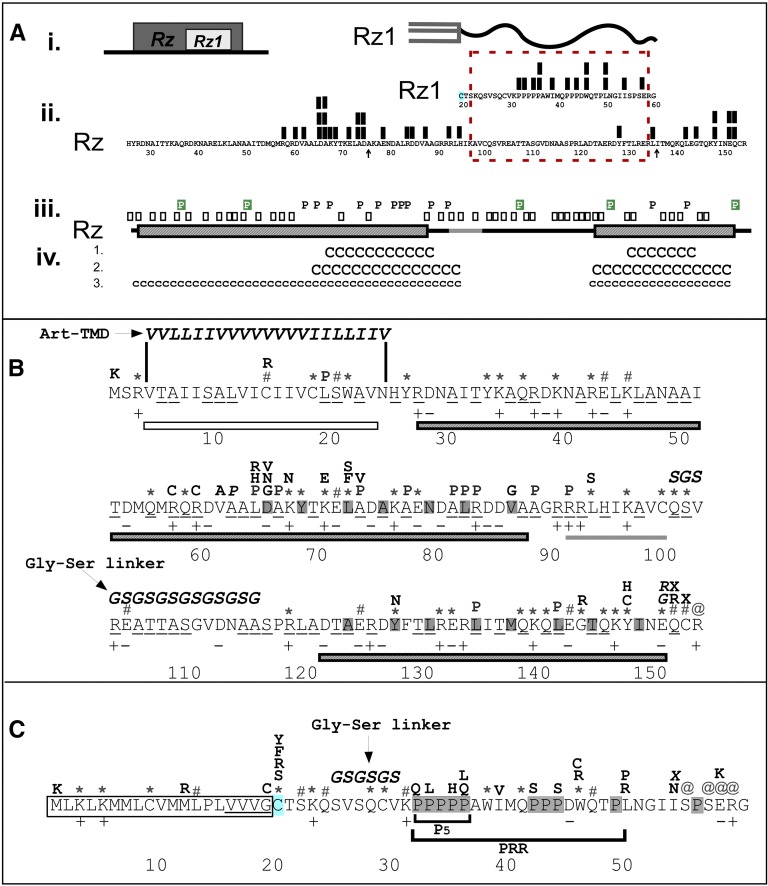
The location of single missense mutants aligned within Rz and Rz1. (A) (i) Rz1 is embedded within Rz. (ii) The coding sequences and cartoon products of *Rz* and *Rz1* are aligned according to embedded architecture. Arrows denote where *Rz1* is embedded within the *Rz* coding sequence. The Rz TMD (1–24) and Rz1 signal sequence (1–19) are removed for clarity. The location of single missense mutants that abrogate function are represented with black boxes above the Rz and Rz1 aa sequence. The lipoylated cysteine of Rz1 (Cys20) is highlighted in blue. The dashed red box highlights a region in the spanin coding sequence where single mutants signal was high in *Rz1*, and low in *Rz*. (iii) Predicted Rz secondary structural features aligned with the primary structure features above. Black line: regions with no predicted secondary structure, Striped rectangles, Rz α-helices; Gray line, β-sheet. Boxes represent *Rz* codons that can be mutated to encode proline in one mutational step. Black “P” denotes positions where a proline blocked function. Green-highlighted “P” denotes positions where a proline mutation did not block function. (iv) Coiled-coil score predictions aligned respective to the secondary structure features of Rz above. (1). “C” represents coiled-coil motif predictions by Coils (score of >0.74, window 14). (2). “C” represents coiled-coil predictions by Coils (score of >0.5, window 21) (3). “c” represents coiled-coil predictions by Paircoil2 (*p*-scores <0.04). (B) Single mutants of Rz shown with primary structure. Single missense mutants are aligned above the amino acid sequence in their respective positions. “*” indicate nonsense codons identified by the screen that were generated by a single base pair change. “#” indicates positions which nonsense codons could be generated with a single base pair change, but were not obtained in the selection. “@” indicates codons of the latter type (#) that do not block Rz function. Underlined residues in the Rz sequence can be changed to Pro with a single base change. Residue charges are identified below the amino acid sequence. Italicized letters indicate mutants identified by site-directed mutagenesis. Relative positions of the Art-TMD, and the Gly-Ser linker are identified with an arrow. The open rectangle, striped rectangle, and gray line represent predictions of the Rz TMD, α-helices, and β-sheet, respectively. The *a* and *d* positions of the Rz coiled-coil as predicted by COILS (score >0.5; window = 21) that fall within the range of predicted Rz α-helix are indicated by gray highlight. (C) The primary structure of Rz1. Single missense mutants are aligned above the amino acid sequence in their respective positions. “*” indicate nonsense codons identified by the screen that were generated by a single base pair change. “#” indicates positions at which nonsense codons could be generated with a single base pair change, but were not detected. “@” indicates codons of the latter type (#) that do not block Rz1 function. Italicized letters indicate mutants identified by site-directed mutagenesis. Relative positions of the Gly-Ser linker are identified with an arrow. The first 19 residues of Rz1 are boxed to represent the signal sequence. The Rz1 lipobox is highlighted at the −1 to −3 positions relative to the lipoylated Cys (20), which is highlighted in blue. Proline residues are highlighted in gray. P_5_ and PRR represent the pentaproline stretch and proline-rich region, respectively.

**Figure 3:**
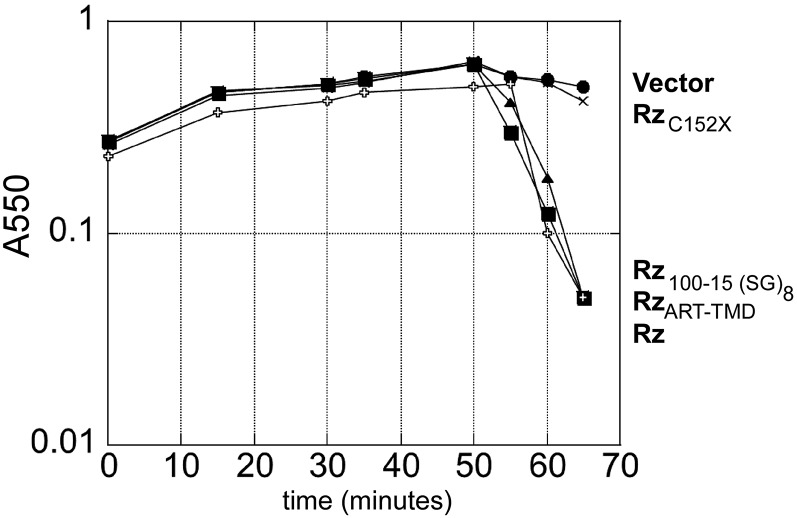
Lysis profile of Rz artificial TMD, artificial linker, and C152X. The following lysogens were induced at time = 0 and monitored at A550: MC4100 (λ900 *Rz_am_*) lysogens carrying either an empty pRE plasmid (vector), or pRE with following *Rz* alleles: pRz (WT), or pRz with an artificial transmembrane domain (ART-TMD), pRz 100–115 (Ser-Gly)_8_, and Rz C152X.

Among the 131 lysis-defective *Rz* alleles obtained by random mutagenesis, 82 alleles were nonsense changes in 34 different positions scattered throughout the periplasmic domain (L1, CC1, L2, CC2, and CTD). Estimates of the Rz-Rz1 complex approximate the span of the periplasm: 170 residues (130 Rz and 40 Rz1) × 0.15 nm/residue, assuming α-helical structure, equals 25.5 nm (Branden 1999). Therefore, it is reasonable to assume that most, if not all, *Rz* nonsense mutations would be lysis-defective. Since there are only 45 positions where nonsense codons can be obtained by a single base change, the random mutagenesis was estimated to be approaching saturation (34 obtained out of 45). The nonsense mutations were distributed across the entire length of Rz, except for the extreme C-terminal region, suggesting that this domain of Rz is not essential (Figure 2B and Table 3). To test this hypothesis, site-directed mutagenesis was used to introduce nonsense mutations in the last three residues. Phenotypic analysis revealed that only the C-terminal R153 residue is dispensable; nonsense mutations at positions 151 and 152 were lysis-defective (Figure 3 and Table 3). Importantly, the C152S allele, which abrogates one of the two intermolecular disulfides of Rz, is functional if Rz1 retains its intermolecular disulfide linkage at C29 (Berry *et al.* 2013). Thus, oddly, Rz can tolerate a Cys to Ser substitution that abrogates the C-terminal disulfide bond, but not a deletion of this residue, suggesting a strict chain length requirement for the Rz periplasmic domain.

**Table 3:** List of *Rz* and *Rz1* mutants grouped by their substructural regions

Rz Mutants					Rz1 Mutants				
Codon	Change	Isolates	Lysis	Region	Codon	Change	Isolates	Lysis	Region
**1**	**Met → Lys**	**1**	**−**	TMD	**1**	Met → Lys	**1**	**−**	Signal sequence
**4**–**24**	**Artificial TMD**	**0**	**+**		**12**	**Met** » **Arg**	**3**	**−**	
**14**	**Cys → Arg**	**2**	**−**		**19**	**Gly** » **Cys**	**3**	**−**	
**19**	**Leu → Pro**	**2**	**−**		**20**	**Cys** » **Ser**	**5**	**−**	
**20**	**Ser → Pro**	**0**	**+**		**20**	**Cys → Arg**	**1**	**−**	
**36**	**Gln → Pro**	**0**	**+**	CC1	**20**	**Cys** » **Phe**	**2**	**−**	
**50**	**Ala → Pro**	**0**	**+**		**20**	**Cys → Tyr**	**3**	**−**	L
**57**	**Arg → Cys**	**2**	**−**		**25**–**30**	**(Gly-Ser)_3_**	**0**	**+**	
**59**	**Arg → Cys**	**1**	**−**		32	Pro » Gln	**1**	**−**	
61	Val **→** Ala	**1**	**−**		33	Pro **→** Leu	**1**	**−**	
62	Ala → Pro	**0**	**−**		35	Pro » His	**3**	**−**	
64	Leu » His	**2**	**−**		36	Pro » Gln	**3**	**−**	
**64**	**Leu → Pro**	**3**	−		**36**	**Pro → Leu**	**2**	**−**	
64	Leu » Arg	**1**	−		**39**	**Ile → Val**	**1**	**−**	Proline-rich-region
65	Asp **→** Gly	**2**	−		**42**	**Pro → Ser**	**1**	**−**	
**65**	**Asp → Asn**	**1**	−		44	Pro **→** Ser	**1**	**−**	
**65**	**Asp** » **Val**	**1**	**−**		**45**	**Asp → Lys**	**0**	**+**	
**66**	**Ala** » **Pro**	**1**	**−**		46	Trp » Cys	**1**	**−**	
**67**	**Lys** » **Asn**	**1**	**−**		46	Trp **→** Arg	**2**	**−**	CTD
**70**	**Lys → Glu**	**1**	−		**50**	**Leu** » **Arg**	**1**	**−**	
72	Leu » Phe	**1**	−		50	Leu **→** Pro	**7**	**−**	
**72**	**Leu → Ser**	**1**	−		54	Ile » Asn	**1**	**−**	
**73**	**Ala** » **Pro**	**1**	**−**		**54**	**Ile → X_(Ochre)_**	**0**	**−**	
73	Ala **→** Val	**1**	−		**55**	**Ser → X_(Amber)_**	**0**	**+**	
**77**	**Ala** » **Pro**	**1**	−		**56**	Pro → X_(Opal)_	**0**	**+**	
**82**	**Leu → Pro**	**1**	−		**57**	Ser → X_(Ochre)_	**0**	**+**	
**83**	**Arg** » **Pro**	**1**	**−**		58	Glu → Lys	**1**	**−**	
86	Val » Gly	**1**	**−**		**58**	**Glu → X _(Ochre)_**	**0**	**+**	
**88**	**Ala** » **Pro**	**2**	−		**59**	**Arg → Glu**	**0**	**+**	
91	Arg » Pro	**2**	−	L1	**59**	**Arg → X _(Ochre)_**	**0**	**+**	
93	Leu **→** Ser	**3**	−		**60**	**Gly →** **X _(Ochre)_**	**0**	**+**	
**100–115**	**(Gly-Ser)_8_**	**0**	+					
**107**	**Thr → Pro**	**0**	+					
**125**	**Arg → Pro**	**0**	+					
127	Tyr » Asn	**1**	−	CC2				
**134**	**Leu → Pro**	**3**	−					
**141**	**Leu → Pro**	**1**	−					
143	Gly » Arg	**1**	−					
**147**	**Tyr → His**	**1**	−					
147	Tyr **→** Cys	**2**	−					
**150**	**Glu → Arg**	**0**	−					
150	Glu → Gly	**0**	−					
151	Gln **→** Arg	**2**	−					
**151**	**Gln** **»** **Lys**	**1**	−					
151	Gln → Pro	**0**	+	CTD				
151	Gln → X _(Ochre)_	**0**	**−**					
152	Cys → X _(Ochre)_	**0**	**−**					
**153**	**Arg → X _(Ochre)_**	**0**	**+**					

The types of residue change, number of isolates obtained by random mutagenesis (positive integers), lysis function, and the relevant structural region of the mutant position are indicated in the table. A type of substitution between residue is indicated by an arrow symbol (→) for transition and by a double greater sign (») for transversion. Mutants indicated in bold were tested for their dominance in the presence of the corresponding wild-type allele. The ability of each allele either to support or block lysis by complementation is indicated by “+” or “**−**” symbol, respectively. Mutants created by site-directed mutagenesis are indicated by a “0” isolate number. Nonsense mutations are indicated by “X” and their type in parenthesis.

#### Lysis-defective missense alleles of Rz:

The remaining 49 alleles were single missense changes in 34 positions in various domains of Rz (Figure 2A), some of which were isolated multiple times, another indication that the selection was approaching saturation (Table 3). Overall, the missense mutations were significantly biased toward the distal half of CC1 (positions 61–87), where ∼62% of the missense changes were clustered in only 20% (residues 57–88) of Rz. The mutations were more prevalent in CC1 (25 alleles in 17 positions) compared to CC2 (six alleles in five positions).

Coiled-coil domains are composed of a heptad repeat motif that typically contains bulky hydrophobic residues alternating at every first and fourth residue (the “*a*” and “*d*” positions of *abcdefg*) (Berger *et al.* 1995; Lupas *et al.* 1991). Six missense alleles were isolated in the eight “*a*” and “*d*” positions between residues 60 and 86 in CC1. Of these, three were conservative mutations (V61A, A73V, and V86G), which cause changes in the bulk of a hydrophobic side chain. This suggests that these residues are involved in interhelical packing of two parallel CC1 domains. One stretch of 10 residues, L64–A73, was the most mutationally sensitive region of Rz, with 13 missense alleles mapping to seven of the 10 positions, including five missense mutations at two “*a*” positions. Within this stretch, L64 and D65 were the most mutationally sensitive positions, with six missense alleles conferring a lysis defect. Although polar or charged residues are tolerated in *a* and *d* positions, it is unusual for Asp to be in the “*a*” position of a parallel helical interface (Akey *et al.* 2001; Parry 1982). It has been reported that Asp (not Glu) in the “*a*” position of a parallel coiled-coil creates a flexible hinge (Straussman *et al.* 2007). Detection of three alleles at this position suggests D65 may form a junction in CC1 that is essential for spanin function.

Interestingly, when coding sequences are aligned respective to the embedded architecture of *Rz* and *Rz1*, there was virtually no overlap between the mutationally sensitive regions of the two genes (Figure 2A, ii, red rectangle), despite the fact that the mutational selections were done separately on each spanin subunit gene, with the cognate subunit supplied *in trans*. Only one missense change, Y127N, was obtained in this region (Figure 2B, positions 94–134). This allele product does not accumulate indicating it is unstable (data not shown). The part of the *Rz1* reading frame encoding the mature lipoprotein lies entirely within this ∼40 codon region of *Rz* that is mutationally silent. Presumably this reflects the unique evolutionary pressures extant in the embedded character of these two genes, so that no part of the nested architecture is subject to the functional requirements of both spanin subunits. This would suggest that i-spanin genes from phages with separated architectures may be free to evolve a more structurally-defined L2 region.

In contrast to the rich and diverse mutational profile of the middle region of CC1, the periphery of CC1 and the entire CC2 domain were relatively insensitive to missense changes other than helix-breaking Pro substitutions. Most of the mutations within CC2 were located at the extreme C-terminus, between residues 143 and 151, with four alleles in three positions. This finding, along with the results of site-directed mutagenesis (see below) suggests this segment of CC2 interacts with Rz1.

#### Phenotypic analysis of proline substitution highlights essentiality of coiled-coil structure within Rz helices:

**Figure 4:**
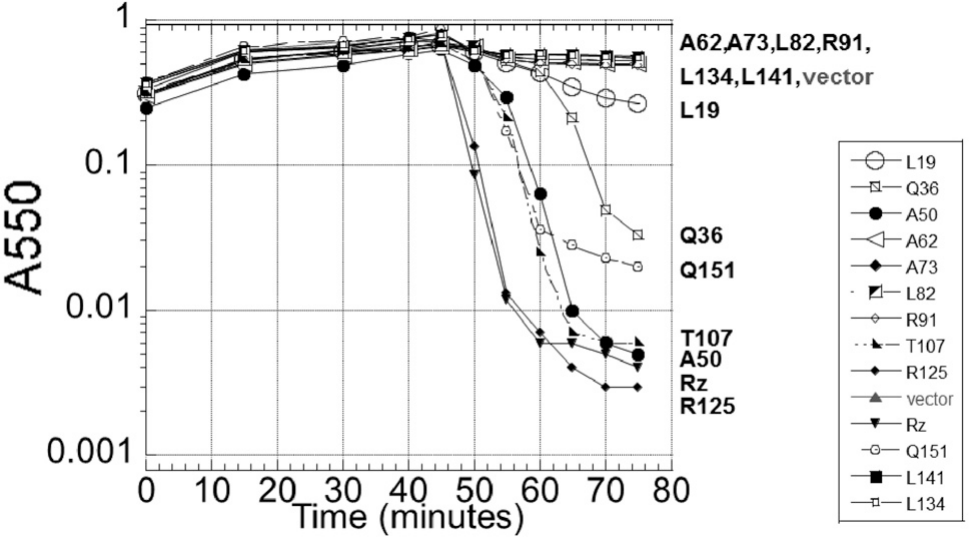
Lysis profile of Rz proline substitutions. The following lysogens were induced at time = 0 and monitored at A550: MC4100 (λ900*Rz_am_*) lysogens carrying either an empty pRE plasmid (vector), or pRE with following *Rz* alleles: pRz (WT), or pRz with the residue and position of proline substitution. The residue and position of the proline substitution within the plasmid-expressed *Rz* allele is identified in the legend.

Of the 34 missense mutations in Rz, 11 were Pro substitutions, including 10 in the predicted coiled-coil helices, and one in the TMD near the periplasmic interface. Given the degree of saturation, the distribution of Pro substitutions within the set of codons that can be changed to a Pro codon with a single base change (*i.e.*, XCX or CXX) should be a good indicator of essential helical secondary structure. We used the One SNP Away tool (Mijalis and Holt 2016) to scan for such codons (Figure 2A, iii). In our screen, we did not isolate Pro substitutions within 20 accessible codons between positions 23 and 63 (*i.e.*, all of L1 and the proximal half of CC1) identified by the screen. Additionally, no prolines were identified in 22 such codons from positions 92–133. Conversely, proline substitutions were isolated in nine of 13 possible positions between 64 and 93. Similarly, there were two of five possible proline substitutions identified within an eight-residue stretch of CC2. Assuming proline substitutions obtained by this selection serve as an indicator of essential helical structure, the essential Rz helices span from position ∼60 to ∼90 and ∼130 to ∼140. Using JPRED4, predictions based on primary structure find longer helices, from 27 to 87, and 121 to 150 (Drozdetskiy *et al.* 2015). To gain more insight to the potential length of these helices, we selected residues Q36, A50, A62, T107, R125, and Q151 for proline substitution (Figure 2A, iii, *cf*. green and black “P” and Figure 4). Among these changes, mutations within the most stringently predicted coiled-coil stretches (Figure 2A, iv: #1 and 2) resulted in lysis-defective alleles. Conversely, residues T107 and Q151 tolerate proline substitution, which would be expected since they are outside of predicted helices. Although Q35, A50, and R125 fall within predicted helices, proline substitutions in these positions do not inactivate Rz function, suggesting that the regions found to be proline-sensitive correspond to coiled-coils. Since these residues sample helical segments of Rz with low scoring coiled-coil prediction, it is apparent that proline substitutions are tolerated only in stretches of Rz without well-defined coiled-coil helical structure. Taken together, these data suggest that CC1 and CC2 are two regions of coiled-coil structure important for spanin function.

#### Role of the Rz TMD:

There were only three mutations isolated in the TMD, suggesting that the TMD serves only as a membrane anchor. We tested this notion by replacing residues 5–24 with an artificial TMD (Figure 2B). The resulting allele was fully functional (Figure 3); however, one missense allele, L19P, was isolated in the lysis-defective selection. Proline residues are generally well-tolerated in TMDs, and would not be expected to abrogate membrane-anchoring (Brandl and Deber 1986; Ulmschneider and Sansom 2001). Furthermore, of seven possible changes to proline accessible by a single-base change in the TMD region, only L19 was isolated. The codon nearest to L19 susceptible to Pro substitution with a single base change is S20; S20P was found to be functional (data not shown). In another type-II membrane protein system, the position of the proline within the TMD affected integration into the membrane, with drastic differences in protein accumulation and maturation observed between adjacent mutated positions (Chung *et al.* 2011). Thus, the proline substitution at position 19 may disrupt function by blocking proper maturation of Rz.

#### The Rz L2 region functions as an unstructured hinge:

Rz has a predicted unstructured region between the CC1 and CC2 (L2 region, positions 89–120). The only L2 lysis-defective mutants isolated, R91P and L93S, are within a predicted β-strand near CC1 (Figure 2, A ii and B). Based on these data and class-I viral fusion models (Kielian 2014; Podbilewicz 2014), we hypothesized that this region functions as a flexible linker to connect two helical domains. To test our hypothesis, residues 100–115 were replaced by a 16-mer consisting of repeats of the Ser-Gly dipeptide sequence, corresponding to Gly-rich flexible spacers that connect domains of multi-domain proteins (Reddy Chichili *et al.* 2013) (Figure 2B). As expected, the synthetic linker replacement did not abrogate spanin function (Figure 3). This supports the notion that the linker region of Rz acts as a hinge to bring the two helical domains of Rz into close proximity, thus resembling canonical membrane fusion systems, where two coiled-coil structures bring the membrane bilayers into close proximity (Rajaure *et al.* 2015; Harrison 2008).

### Mutational analysis of Rz1

#### Lysis-defective mutants of Rz1:

Of a total of 115 lysis-defective *Rz1* mutants, 79 had single point mutations; the rest had two or more mutations or frameshift mutations, and were excluded from analysis. Initially, the degree of saturation was thought to be less than that obtained for *Rz*, because the 36 nonsense mutations were found in only 10 of the 19 codons for which a single nucleotide change could yield a stop codon. However, four such codons were in the CTD of *Rz1*, beyond the last nonsense mutation (W46X) that was obtained in the lysis-defect selection. This raised the possibility that the extreme C-terminus of Rz1 is dispensable. This notion was confirmed when each of these six distal sites was converted to nonsense codon by site-directed mutagenesis, and tested for their function. None were found to have a lysis defect (Table 3). Thus, 10 of 14 potential nonsense sites were accessed in the selection, indicating the degree of saturation was similar to that obtained for *Rz*. The 43 lysis-defective alleles with single missense mutations mapped to only 14 codons of the 60 codons of *Rz1*, and seven of these mutations mapped to four positions in the signal sequence, all of which would abolish translation or processing of the precursor (Von Heijne 1985; Narita and Tokuda 2010). The 36 missense mutations in the periplasmic domain mapped to only 11 positions, none of which were in the first 10 residues of the periplasmic domain. Taken with the nonessential character of the extreme C-terminus, these results indicate that the central 57% (residues 32–54) of the periplasmic domain comprises the key functional domain of Rz1.

#### Rz1 also has a periplasmic linker:

Since no lysis-defective missense mutations were mapped in the first 11 residues of the periplasmic domain, we hypothesized that the N-terminal segment of the mature periplasmic domain of Rz1 could function as a flexible spacer between the membrane-attached N-terminus and the mutationally sensitive central domain, like the linker region between the coiled-coil domains in Rz. When we replaced residues 25–30 with three Gly-Ser repeats (Figure 2C), the substitution allele was found to retain lytic function, supporting the notion that the role of this region is to link the central domain to the lipid anchor in the inner leaflet of the OM. It should be noted that the linker substitution also abolished the intermolecular disulfide link at position 29, which would disrupt the homodimerization of Rz1. However, this is consistent with previous findings, since spanin function is retained unless homotypic intermolecular disulfide bonds at both Rz1_C29_ and Rz_C152_ are disrupted (Berry *et al.* 2013).

#### The proline rich region of Rz1 is an essential fusion motif:

A striking feature of the mutational distribution, in contrast to the frequency of mutations to proline in *Rz*, is the prevalence of mutations in the Pro codons of *Rz1*. Rz1 is proline-rich, with 10 Pro residues occupying 25% of the mature sequence. Most mutants were within the Proline-Rich Region (PRR) (Figure 1C), especially in four Pro residues in a penta-proline (P_5_) stretch (Figure 2C), residues 32–36. Interestingly, within P_5_, position 34 was not sensitive to alanine replacement, consistent with our previous finding, where an alanine substitution at position 34 did not abrogate spanin function (Berry 2011).

Another lipid-anchored peptide with proline-rich motifs is the reovirus p15 fusion-associated small transmembrane (FAST) protein (Top *et al.* 2012). Similar to the P_5_ stretch of Rz1, p15 has a proline stretch (PPAPPP). Like Rz1, the proline-rich motif in p15 is important for membrane fusion, and the fusion reaction is not sensitive to changes in the third position. Evidence has been presented that the role of polyproline helices in membrane fusion is to promote exposure of hydrophobic side chains of neighboring regions (Top *et al.* 2012).

Mutations in four other positions in the periplasmic domain of Rz1 were lysis-defective: I39V, W46R, W46C, L50P, L50R, and I54N. Of these mutations, the I39V is the most remarkable; Ile and Val side-chains are extremely similar in most contexts except for helix–helix packing (Zhu *et al.* 1993), suggesting that position 39 is involved in an intimate protein–protein contact required for spanin function. The Cys substitution at position 46 (W46C) would be predicted to result in an intramolecular disulfide bond with C29, placing a covalent constraint on the folding of Rz1 (Berry *et al.* 2013). Interestingly, although change-from-proline mutations dominate the mutational spectrum of *Rz1*, L50P, which creates Pro–Pro sequence in the distal region of Rz1 blocks function. This suggests a Pro-sensitive secondary structure, presumably α-helix, is required at the C-terminus.

### Phenotypic analysis of Rz and Rz1 lysis-defective mutants

#### Accumulation of Rz and Rz1 mutant gene products:

**Figure 5:**
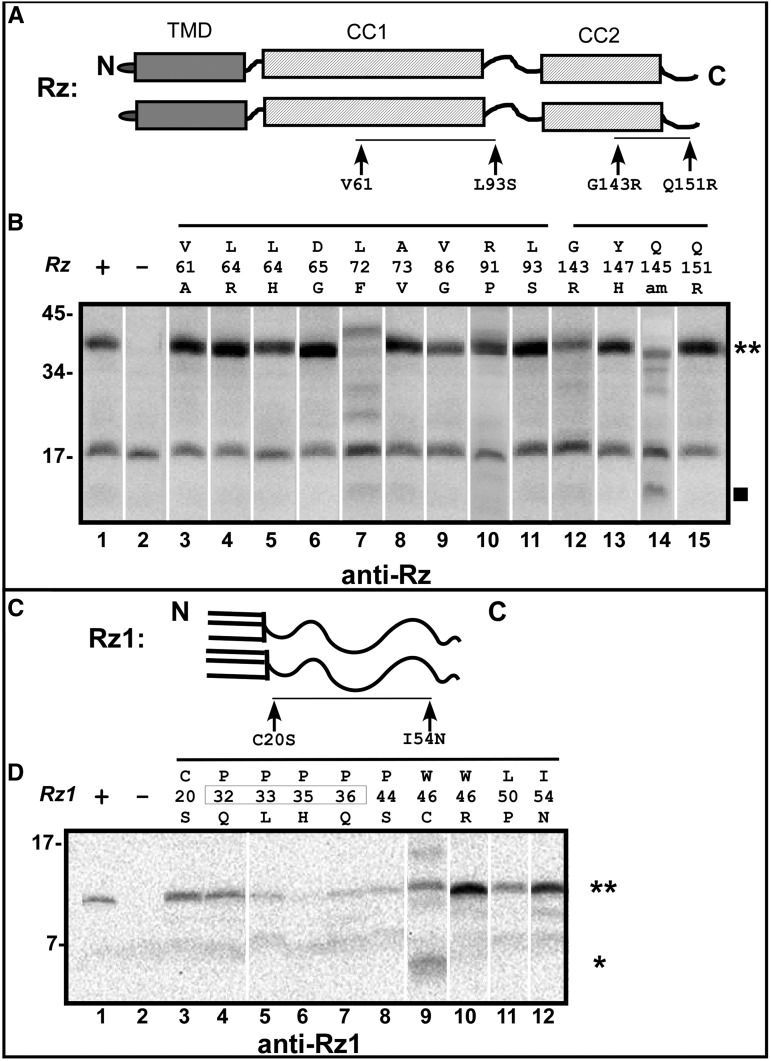
Accumulation of spanin mutant gene products. (A) A cartoon of the Rz dimer is shown. Arrows indicate the positions of V61, L93, G143, and Q151 with respect to predicted features of Rz. The black line between the arrows indicates the relative position of mutants in (B) analyzed by the Western Blot. (B) Anti-Rz Western Blot of Rz mutants in the presence of Rz1. Rz mutants are identified above each lane. The Rz dimer band is denoted by “**,” and the Rz breakdown product is denoted by the square symbol. (C) A cartoon of the Rz1 dimer is shown. Arrows indicate the positions of C20 and I54N. The black line between the arrows indicates the relative position of mutants in (D) analyzed by the Western Blot. (D) Anti-Rz1 Western Blot. Rz1 mutants are identified above each lane. The Rz1 dimer band is denoted by “**,” and the Rz1 monomer product is denoted by “*.”

To determine whether the lysis-defect of the missense mutations reflected a lack of accumulation of either spanin subunit, we collected whole-cell samples of cells expressing plasmid-borne *Rz* or *Rz1* mutants in the presence of Rz1 or Rz, respectively. Samples were collected before lysis by TCA precipitation, and examined by Western blotting (see *Materials and Methods*). Most of the allele products accumulated to wild-type levels, indicating that the lysis defect is not due to protein synthesis or stability (Figure 5). For Rz, L72F, G143R, and Q145 am appeared to be unstable, marked by reduced accumulation, and the presence of apparent degradation products (filled square in Supplemental Material, Figure S1) or smears. Surprisingly, in the presence of Rz1, Rz_G143R_ is stabilized and Rz_L72F_ is unstable. We interpret this as evidence that Rz and Rz1 form a complex *in vivo* during the late gene expression period, and mutant products are stabilized, in the case of Rz_G143R_, or destabilized (in the case of Rz_L72F_) by conformational changes associated with complex formation. The accumulation of Rz1_P33L_, Rz1_P35H_, and Rz1_P36Q_ appeared diminished, but these mutations fall within the epitope used for immunodetection, so it is unclear if accumulation is actually affected. Importantly, almost all *Rz* and *Rz1* mutant products appear to accumulate exclusively as disulfide-linked homodimers (double asterisks), with the exception of Rz1_W46C_, which likely is largely blocked in an internal disulfide-bonded state (single asterisk in Figure 5D). Thus, the function of these defective alleles is likely blocked after their dimerization step (Berry *et al.* 2013).

#### Assessing interaction between Rz and Rz1:

**Figure 6:**
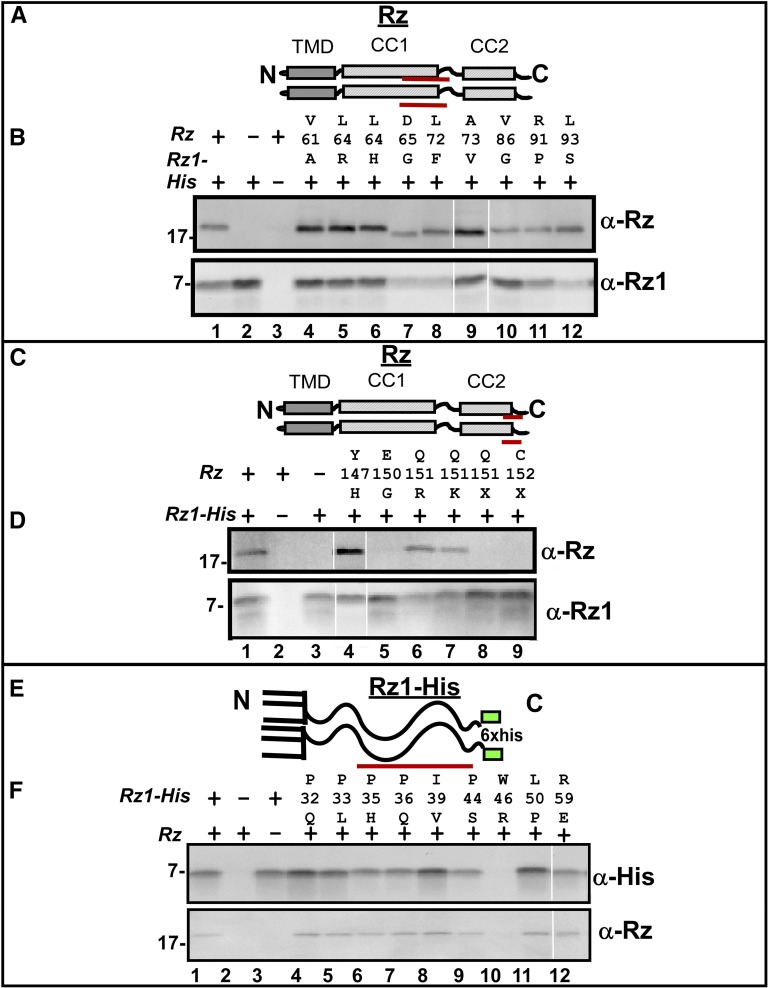
Oligohistidine pulldown of spanin mutants. (A) Cartoon of Rz structure showing the relative location of CC1 mutants used in the oligohistidine pulldown. The TMD, and proximal and distal helices are represented with gray and striped boxes, respectively. Red lines below CC1 represent the relative position of mutant residues used in the pulldown assay. (B) Coimmunoprecipitation of Rz CC1 mutants with Rz1-His by oligohistidine pulldown. Pulldown products were analyzed by Western blot with anti-Rz and anti-Rz1 antibodies. (C) Cartoon of Rz structure showing the relative location of CC2 mutants used in the oligohistidine pulldown. The TMD, and proximal and distal helices are represented with gray, and striped boxes, respectively. Red lines below CC2 represent the relative position of mutant residues used in the pulldown assay. (D) Coimmunoprecipitation of Rz CC2 mutants with Rz1-His by oligohistidine pulldown. Pulldown products were analyzed by Western blot with anti-Rz and anti-Rz1 antibodies. (E) Cartoon of Rz1-His showing relative location of mutants used in the oligohistidine pulldown. Red lines below Rz1-His represent the relative position of mutant residues of Rz1-His used in the pulldown assay. This position of the His tag is represented with a green box. (F) Coimmunoprecipitation of Rz1-His mutants with Rz. Pulldown products were analyzed by Western blot with anti-Rz and anti-Rz1 antibodies.

To test if the various Rz mutants were able to interact with wild type Rz1, we used a pull-down approach with a functional oligohistidine-tagged Rz1, as described before (Berry *et al.* 2008). Preliminary data suggested that a majority of Rz mutants coexpressed with Rz1-His were not defective in coimmunoprecipitation. To increase stringency of the assay, each spanin subunit was expressed in separate cultures before interrogating complex formation *in vitro* with solubilized samples. Nine mutant alleles mapping to CC1 were tested, and, in each case, the Rz product was found to copurify with Rz1-His (Figure 6, A and B), suggesting the defect imposed by substitutions at CC1 does not alter Rz–Rz1 interaction. These lysis-defective alleles of *Rz* and *Rz1* were also tested for dominance by expressing *Rz* or *Rz1* mutants from the pRE plasmid in the presence of a prophage-borne wild-type copy of *Rz* or *Rz1*. All of the mutant alleles tested for complex formation were also unable to block lysis (marked bold in Table 3). The absence of dominant negative character suggests either that (1) there are enough mutant-free spanin complexes present to achieve lysis, or (2) hybrid complexes are not poisoned by the presence of mutant product(s).

**Figure 7:**
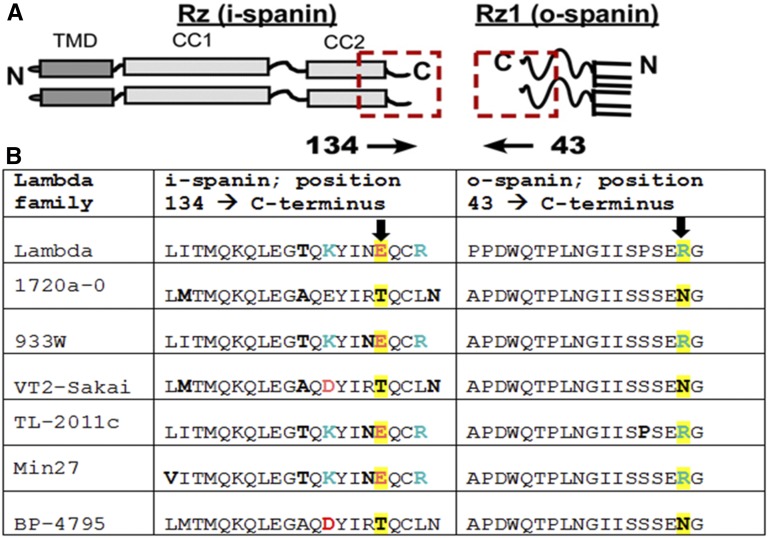
Covariance of Rz E150 with Rz1 R59. (A) A cartoon model of Rz and Rz1 positioned with C-termini in apposition. The red square highlights the relative position of the amino acid sequences used for covariance analysis. (B) Six lambda family spanin equivalents are aligned respective to position 134–153 of Rz and 43–60 of Rz1. To match the cartoon above, the Rz1 sequence is arranged C–N terminus (positions 60–43). Red and Blue letters identify positions with changing positive and negative charge. Bolded letters identify positions with changes to polar residues. The highlighted positions correspond to Rz E150 and Rz1 R59.

**Figure 8:**
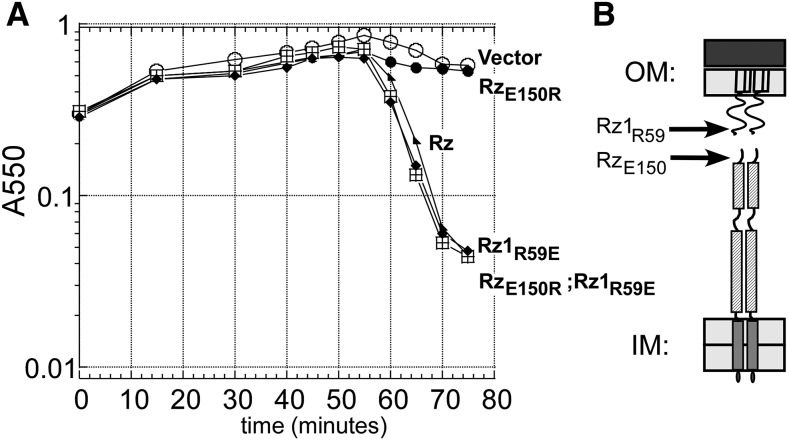
Lysis profile showing Rz1 R59E suppresses the Rz E150R defect *in vivo*. (A) The following lysogens were induced at time = 0 and monitored at A550: MC4100 (λ900 *Rz_am_*) carrying the following plasmids pRE (open circle), pRE Rz (triangle), pRE Rz E150R (closed circle). MC4100 (λ900 *Rz_am_ Rz1_am_*) carrying pRE Rz E150R Rz1 R59E (square), MC4100 (λ900 *Rz1_am_*) carrying pRE Rz1 R59E (diamond). (B) Cartoon of the spanin complex in the cell envelope. The relative positons of Rz E150 and Rz1 R59 are identified by arrows. IM, inner membrane; OM, outer membrane.

To address the C-terminal residues involved in Rz–Rz1 interaction, we used the pull-down assay to characterize six mutants in CC2, including three (Y147H, Q151R, and Q151K) identified by the screen, and three alleles created by site-directed mutagenesis (E150G, Q151X, and C152X). The only CC2 mutant that did not copurify with Rz1-His was Rz_E150G_, suggesting this terminal Glu provides an anionic interaction partner with Rz1 (Figure 6, C and D). The Rz_Q151_ and Rz_C152_ nonsense mutants are defective in accumulation, independent of coexpression with Rz1 (Figure S2), suggesting these mutants are defective in complex formation *in vivo*. Since abrogating negative charge at 150 blocked complex formation *in vitro*, we examined covariance at the C-termini of Rz and Rz1 equivalents in other lambdoid phages (Figure 7). This analysis suggest a linkage between Rz_E150_ and Rz1_R59_; charge-to-polar changes in position E150 are compensated by complementary changes at R59 (Figure 7B). To address whether an electrostatic interaction was required between the residues at this position, we tested whether the *Rz1*_R59E_ allele would suppress the *Rz*_E150R_ defect. Indeed, coexpression of Rz1_R59E_ and Rz_E150R_ complemented the lysis defect of phages carrying *Rzam*/*Rz1 am* *in vivo* (Figure 8). This strongly supports the notion that the heterotypic Rz-Rz1 interaction involves a salt bridge between Rz_E150R_ and Rz1_R59E_.

To screen Rz1 mutants for interaction defects, we created nine mutant alleles of *Rz1-His*. Similar to Rz CC1 mutants, all products tested from *Rz1-His* mutants exhibited parental coimmunoprecipitation with Rz (Figure 6, E and F), suggesting that such mutants are not defective in forming an Rz-Rz1 complex, and are presumably defective in a step following complex formation, *i.e.*, the fusion step(s).

As noted above, Rz1 could be truncated to position 55 without loss of function (Table 3). This is surprising because Rz1 S55X eliminates the C-terminal residues from Rz1, including the salt bridge between Rz1 59 and Rz E150. The simplest explanation is that there are more than one residue pairs involved in Rz-Rz1 complex formation. An overdetermined interaction interface between Rz and Rz1 would provide multiple points of contact that may stabilize the spanin complex.

### Conclusions: Coiled-coils and prolines—a novel fusion matchup

Here, we report the first genetic analysis of an embedded gene pair, of which both genes are required for the same biological function: *Rz* and *Rz1*, which encode the subunits of the two-component spanin of phage lambda. The selection, based on a near-saturation selection for mutants that abrogated lysis, identified mutants that inactivate either Rz, the i-spanin, or Rz1, the o-spanin product encoded by the embedded gene. The selections were done on artificially disembedded genes, but, despite this architectural segregation, both genes exhibited mutational clustering in regions that corresponded to mutationally silent regions of the out-of-frame gene. These mutationally silent regions were tested by site-directed mutagenesis, and found to be replaceable by simple repeated linker sequences, thereby establishing that both Rz and Rz1 have flexible linker domains between the mutationally sensitive regions. Surprisingly, the mutants that were identified by the selection, despite the loss of lytic function, uniformly maintained the ability to form spanin complexes *in vitro* and *in vivo*, and most were not defective in the accumulation of gene products. The simplest interpretation is that these mutations blocked a step downstream of periplasm-spanning complex formation. We have proposed that the complex, once liberated from the constraints of the intact PG layer, undergoes oligomerization, and then causes fusion between the IM and OM (Rajaure *et al.* 2015). The pattern of disabling missense changes in both Rz and Rz1 is consistent with the notion that most of these mutations block spanin function at this putative fusion step. Importantly, the pattern of single missense mutants highlights mutationally sensitive subdomains that resemble known fusion motifs, such as domains that are rich in coiled-coils and proline. In class I viral fusion systems, coiled-coils promote oligomerization and conformational change from extended to hairpin structure, which pulls membranes into apposition. Future studies of mutant alleles of Rz that fall within the coiled-coil domains could determine whether function loss is at the prehairpin formation, or the subsequent conformational change, step. Another unique feature of the spanin fusion array is the PRR in Rz1. As discussed above, polyproline stretches are key fusion motifs in reovirus FAST fusion proteins. Although there is no robust molecular model for the role of proline-rich stretches in the membrane fusion process, single missense mutants in the PRR region of Rz1 suggest a more specific role than membrane disordering. If the role of the PRR is to force exposure of hydrophobic residues, this may promote fusion by increasing contact between Rz1 and the lipid monolayer. In this way Rz1 could act as a scaffold to promote lipid curvature or to promote stalk radius enlargement, mechanisms which have been proposed in other systems (Jackson and Chapman 2006; Chernomordik *et al.* 2006). It will be important to test these models against PRR mutants by developing an *in vitro* fusion system for the spanins.

The Rz-Rz1 spanin system, with its powerful genetics, may be a useful platform for the study of membrane fusion in general. For example, because the spanin-mediated fusion event would have to occur within a 25 nm space between membranes at a precise time in the infection cycle, it may be possible to capture the hemifusion state *in vivo* by using high resolution cryo-EM, and super-resolution microscopy techniques.

## Supplementary Material

Supplemental material is available online at www.g3journal.org/lookup/suppl/doi:10.1534/g3.116.037192/-/DC1.
