# Supplementary figures and images for: Genetic Analysis of the Lambda Spanins Rz and Rz1: Identification of Functional Domains
Source: G3 (Bethesda). 2016 Dec 28;7(2):741–53. doi: 10.1534/g3.116.037192 (PMC5295617; doi:10.1534/g3.116.037192)

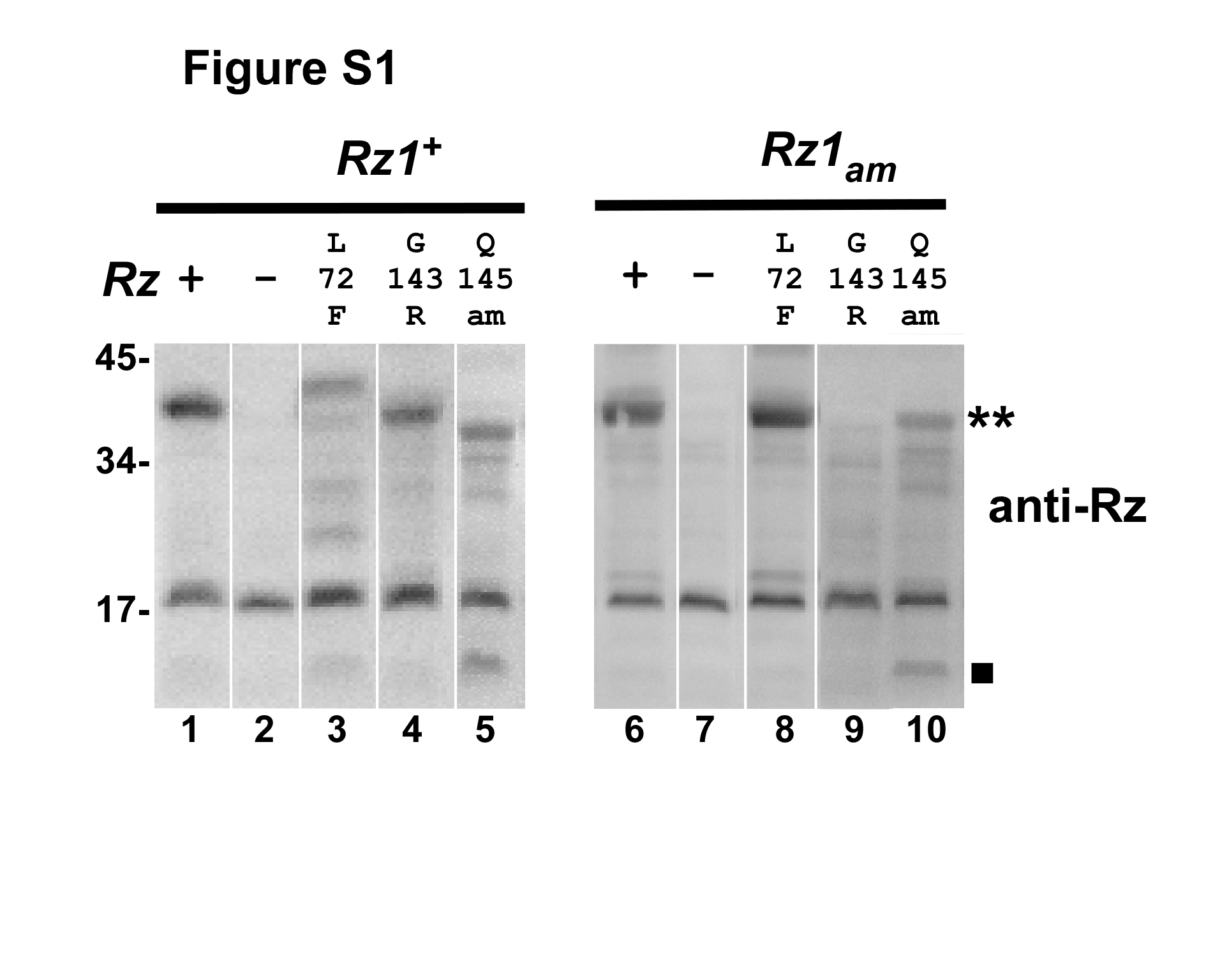

Supplement: Supplementary file 1 [file 741FigureS1.jpg]

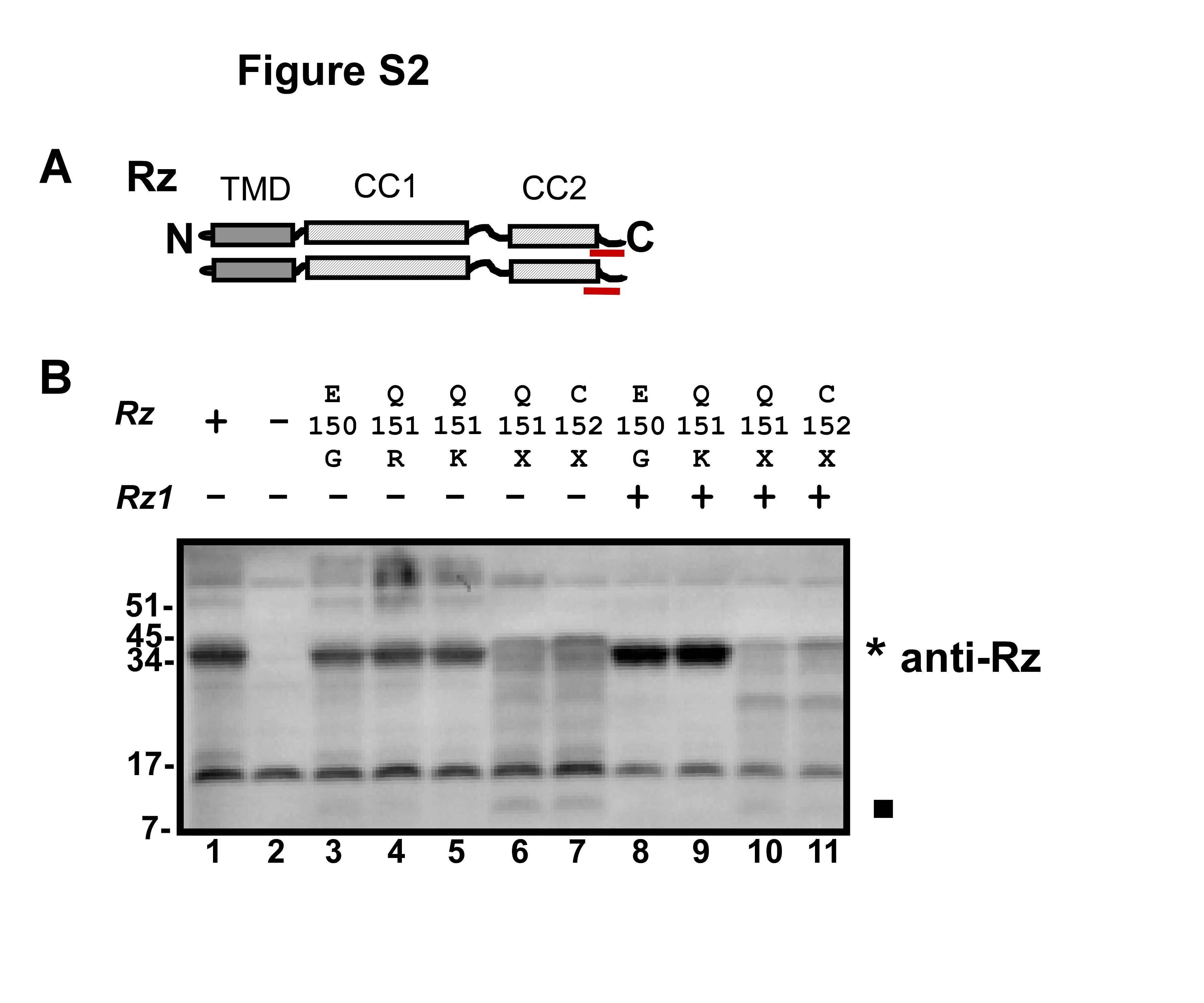

Supplement: Supplementary file 2 [file 741FigureS2.jpg]
